# Pinin Modulation Restores Chemosensitivity in SW620 and HCT-116 Colon Cancer Cells

**DOI:** 10.32604/or.2026.084313

**Published:** 2026-10-28

**Authors:** Alice Molli, Daniela Buonvicino, Gianluca Mattei, Leonardo Bonacchi, Irene Scuffi, Sara Calcagno, Giulia Isoldi, Nicola Schiavone, Francesco De Logu, Alberto Magi, Matteo Lulli, Astrid Parenti, Andrea Lapucci

**Affiliations:** 1 Department of Health Sciences, Section of Clinical Pharmacology and Oncology, University of Florence, Florence, Italy; 2 Unit of Translational and Precision Medicine, Meyer Children’s Hospital IRCCS, Florence, Italy; 3 Department of Experimental and Clinical Biomedical Sciences, “Mario Serio” University of Florence, Florence, Italy; 4 Department of Information Engineering, University of Florence, Florence, Italy

**Keywords:** Colorectal cancer, chemoresistance, RNA processing, oxaliplatin, 5-fluorouracil, metabolic reprogramming

## Abstract

Objectives: Colorectal cancer (CRC) remains a leading cause of cancer-related mortality, largely due to the emergence of resistance to standard chemotherapeutic regimens. The identification of molecular determinants of chemoresistance is therefore critical to improve patient stratification and therapeutic efficacy. Here, we investigate the functional role of Pinin (PNN), a multifunctional protein involved in RNA processing and gene regulation, in mediating chemoresistance in CRC. Methods: SW620 and HCT-116 colorectal cancer cells were used to investigate the functional role of PNN. *Pnn* expression was silenced by siRNA and lentiviral shRNA approaches. Cell sensitivity to 5-fluorouracil and oxaliplatin was evaluated by MTT assay, while apoptosis was assessed by Annexin V/7-AAD flow cytometry. Migration, invasion, and clonogenic capacity were analyzed using wound-healing, modified Boyden chamber, and colony formation assays. Metabolic reprogramming following *Pnn* depletion was characterized using Seahorse extracellular flux analysis. Transcriptomic alterations were investigated by Nanopore RNA sequencing, followed by differential gene expression, bioinformatic, and gene set enrichment analyses. Results: *Pnn* silencing significantly enhanced sensitivity to 5-fluorouracil and oxaliplatin in SW620 cells, as demonstrated by reduced cell viability, decreased IC_50_ values, and increased apoptotic responses. Transcriptomic profiling revealed extensive gene expression reprogramming, affecting pathways related to cell cycle progression, DNA damage response, and stress signaling. To corroborate these findings, *Pnn*-silenced HCT-116 cells were used to confirm the observed cytotoxic effects and independently validate selected differentially expressed genes identified by RNA-seq in SW620 cells. Notably, *Pnn* depletion induced a marked impairment of mitochondrial oxidative phosphorylation without compensatory glycolytic activation, indicating reduced metabolic flexibility under stress conditions. Functionally, *Pnn* silencing also decreased clonogenic capacity and invasive behavior. Conclusion: Collectively, these findings identify *Pnn* as a key regulator of chemoresistance and reveal its involvement in transcriptional control, metabolic adaptation, and stress response. Targeting PNN-related pathways may represent a promising therapeutic strategy to restore chemosensitivity and improve outcomes in colorectal cancer, although further validation is required.

## 1 Introduction

Colorectal cancer (CRC) remains one of the most prevalent and deadly malignancies worldwide, accounting for nearly 10% of all cancer-related deaths annually [1]. Despite significant advances in screening, diagnosis, and multimodal therapeutic strategies, including surgical resection, radiotherapy, and systemic treatments such as chemotherapy, targeted therapy, and immunotherapy, CRC continues to pose a major public health challenge. One of the primary obstacles to effective management is the development of resistance to chemotherapy, which severely limits treatment success and contributes to disease recurrence and metastasis [2]. Over the past two decades, extensive research has focused on identifying the molecular drivers of CRC progression and resistance. Several oncogenic alterations have been implicated, including aberrant activation of KRAS, BRAF, PIK3CA, and S100P [3]. However, the molecular mechanisms underlying migration, invasion, metabolic adaptation, and the acquisition of the chemoresistant phenotype remain incompletely understood. Both intrinsic and acquired resistance arise through a multifactorial process involving tumor heterogeneity, epigenetic remodeling, and complex interactions with the tumor microenvironment. Furthermore, key signaling cascades such as Wnt/β-catenin, PI3K/AKT, MAPK, and TGF-β, along with epithelial–mesenchymal transition (EMT) and cancer stem cell (CSC) dynamics, play essential roles in sustaining tumor cell survival and adaptability under therapeutic pressure. Within this complex biological landscape, the identification of novel molecular regulators that control resistance-associated signaling pathways is crucial to improving treatment efficacy and clinical outcomes. Pinin (PNN) is a 140 kDa phosphoprotein initially characterized as a desmosome-associated component involved in maintaining epithelial cell adhesion and integrity [4]. Subsequently, PNN has been found to localize in the nucleus, where it participates in transcriptional regulation, mRNA splicing, and chromatin organization. This dual localization suggests that PNN acts as a multifunctional scaffold protein integrating cytoplasmic adhesion signaling with nuclear gene regulatory networks. Early studies proposed a tumor-suppressive function for PNN in renal cell carcinoma [5], but more recent findings have revealed its potential oncogenic activity, particularly through the activation of EGFR/ERK and PI3K/AKT signaling pathways [6,7]. This shift in perspective has positioned PNN as a key player in epithelial cell plasticity and tumor progression [8]. PNN has also been implicated in regulating alternative splicing events that influence genes involved in proliferation, apoptosis, and cytoskeletal remodeling, processes intimately linked to cancer progression and drug response. Its contribution to epithelial integrity and cellular stress adaptation suggests that PNN could serve as a molecular switch connecting cell adhesion dynamics with the regulation of survival pathways. Notably, alterations in splicing machinery components have emerged as pivotal mechanisms in tumor adaptation and resistance, supporting the hypothesis that PNN dysregulation may contribute to chemoresistance through splicing-dependent signaling modulation.

To the best of our knowledge, our previous studies provided the first clinical evidence that high PNN expression is associated with poor prognosis in stage III CRC patients treated with adjuvant fluoropyrimidine- and oxaliplatin-based chemotherapy [9]. This finding was subsequently validated in an independent cohort of 74 formalin-fixed paraffin-embedded tumor and matched normal mucosa samples from stage II–III CRC patients receiving adjuvant therapy, confirming the predictive value of *Pnn* mRNA levels for disease-free survival (DFS) [10]. Together, these clinical data suggest that *Pnn* may represent a potential biomarker associated with chemoresistance in colorectal cancer. In the present study, we aimed to elucidate the functional role of PNN in CRC chemoresistance by integrating phenotypic, molecular, transcriptomic, and functional analyses. To this aim, we employed two intrinsically resistant CRC cell line models (SW620 and HCT-116 cells) to assess how *Pnn* silencing affects drug resistance in response to standard chemotherapeutic agents such as oxaliplatin (OHP) and 5-fluorouracil (5-FU).

## 2 Materials and Methods

### 2.1 Cell Culture

The human colorectal cancer cell lines SW620 (CCL-227) and HCT-116 (CCL-247) were purchased from ATCC (Manassas, VA, USA). SW620 and HCT-116 cells were maintained in Dulbecco’s Modified Eagle Medium (DMEM) (Euroclone, ECM0728L, Pero, Italy) supplemented with 10% fetal bovine serum (FBS) (Euroclone, ECS5000L), penicillin (100 U/mL) and streptomycin (100 U/mL) (Euroclone, ECB3001D) at 37°C in a humidified incubator with 5% CO_2_. Cell lines were authenticated by ATCC using short tandem repeat (STR) profiling. Cells were periodically screened for mycoplasma contamination using PCR.

### 2.2 Cell Viability

Cell viability was assessed using the 3-(4,5-dimethylthiazol-2-yl)-2,5-diphenyltetrazolium (MTT) (Merck, 475989, Milan, Italy) assay. Briefly, exponentially growing SW620 or HCT-116 cells were seeded in 96-well plates in complete medium at a plating density of 2 × 10^3^ cells/well (cell confluence was monitored during the experiment to exclude potential effects of overgrowth on the MTT measurements). After 24 h, cells were exposed to increasing concentrations of 5-Fluorouracil (5-FU) (Merck, F6627) (0.1, 0.3, 1, 3, 5, 10, 22, 30, 100 μM for SW620 cells; 0.02, 0.1, 0.5, 1, 3, 10, 30, 100 μM for HCT-116 cells) or Oxaliplatin (OHP) (Merck, O9512) (0.03, 0.1, 0.2, 0.3, 1, 3, 4, 10, 30 μM for SW620 cells; 0.02, 0.1, 0.5, 1, 3, 10, 30, 100 μM for HCT-116 cells). After 72 h, treated cells were incubated at 37°C for 20 min with 1 mM MTT (diluted in Dulbecco’s Phosphate Buffered Saline (PBS) (Euroclone, ECB4004L)). After removal of the medium, cells were exposed to DMSO for 15 min, and the supernatant was collected. Absorbance at 550 nm was measured using a multilabel microplate reader VICTOR3 (Perkin Elmer, 1420-012, MA, USA). All the reported IC50 values represent the means of at least three independent experiments. *t*-Tests were used for pairwise comparisons. *p* values < 0.05 were considered statistically significant. All data were analyzed using GraphPad Prism version 5 (GraphPad Prism Software Inc., La Jolla, CA, USA). IC50 values were calculated using GraphPad Prism v7 software (GraphPad Prism, La Jolla, CA, USA). IC50 drug concentrations were defined as those resulting in a 50% reduction of cell viability of drug-treated cells compared to untreated control cells. All reported IC_50_ values represent the means of at least three independent experiments.

### 2.3 Silencing

SW620 cells were transfected with 100 nM specific siRNA against human *Pnn* or siRNA negative control (IDT, PNN hs.Ri.PNN.13.2 and 51-01-14-04, Leuven, Belgium), using jetPRIME^®^ (Polyplus, 101000015, Illkirch, France) according to the manufacturer’s instructions. The effects of silencing were evaluated 48- and 72-h post-transfection by RT-qPCR and western blot. Furthermore, stable shRNA-Pnn cells were generated by means of lentiviral vectors. SW620 and HCT-116 cells were infected with lentiviral particles carrying either pLV-shRNA-Pnn target sequence 5′-ATACTTCAGGACTAGAAAGAAGTCA-3′ or negative control pLV-shRNA-C target sequence 5′-CCTAAGGTTAAGTCGCCCTCG-3′ (VectorBuilder GmbH, VB260605-1121kus and VB010000-9541pqu seq, Neu-Isenburg, Germany), each vector containing the same nucleotide sequence used in the siRNA silencing experiments. Cells were infected at a multiplicity of infection (MOI) of 10 in the presence of 8 μg/mL polybrene for 24 h. Stable pooled cell populations, rather than individual clones, were generated following puromycin selection (4 μg/mL; Merck, P9620-10mL, Milan, Italy). The effects of Pnn silencing were subsequently evaluated as described above. All silencing validation experiments were performed in at least three independent biological replicates.

### 2.4 RNA Extraction and Quantitative Real-Time PCR (RT-qPCR)

Total RNA was isolated from SW620 cells treated with siRNA against *Pnn* or scrambled siRNA control, or from SW620 and HCT-116 cells treated with pLV-shRNA-Pnn or pLV-shRNA-C control, using the NucleoSpin Total RNA kit, according to the manufacturer’s protocol (Macherey-Nagel, 740955.50, PA, USA). RNA was quantified using a Qubit™ 3.0 Fluorometer (Invitrogen, Q33216 MA, USA) by means of Qubit RNA BR Assay Kit (Invitrogen, Q10210). RNA quality was assessed using the Agilent RNA 6000 Nano LabChip^®^ kit with an Agilent 2100 Bioanalyzer (Agilent Technologies, 5067-1511, CA, USA). Two hundred ng of total RNA was reverse-transcribed using iScript cDNA Synthesis Kit (Bio-Rad, 170-8891, CA, USA) in a total volume of 20 μL, following the manufacturer’s standard protocol, which includes predefined incubation conditions, reaction times, and enzyme amounts. The resulting cDNA was then amplified with specific primers listed in Supplementary Table S1.

PCR amplification was carried out using SsoAdvanced™ Universal SYBR^®^ Green Supermix (Bio-Rad, 1725270, USA) according to the manufacturer’s instructions using a RotorGene 3000 (Qiagen, 9001862, Germany). In this analysis, *18S* rRNA was confirmed as stable internal control. Relative gene expression was calculated using the 2^−ΔΔCt^ method. RT-qPCR was performed using the following cycling conditions: 98°C for 2 min, followed by 40 cycles of 98°C for 5 s and 60°C for 10 s. The program was set to reveal the melting curve of each amplicon from 60°C to 95°C with readings every 0.5°C. All RT-qPCR analyses were performed in at least three independent biological replicates.

### 2.5 Western Blot Assay

For Western blot analysis, cells were lysed in cold RIPA buffer (1% NP-40, 150 mM NaCl, 5 mM EDTA, 1% NaDOC, 25 mM Tris-HCl pH 7.6, 0.1% SDS) (Thermo Fisher Scientific, 89900, Rockford, IL, USA) supplemented with protease and phosphatase inhibitors (Merck, 524624). Briefly, following protein quantification with the BCA Protein Assay (Thermo Fisher Scientific, A55860), aliquots of extracted total protein (60 μg) were electrophoresed on sodium dodecyl sulfate-polyacrylamide gels (SDS-PAGE) with 12% Tris-HCl gels (Bio-Rad Laboratories, 3450014, Hercules, CA, USA). Separate proteins were transferred onto polyvinylidene difluoride (PVDF) membranes (Bio-Rad Laboratories, 1620177). Membranes were blocked for 1 h at room temperature with Intercept blocking buffer (LI-COR Biosciences, Cat. No. 927-70001) diluted 1:1 in PBS (EuroClone, ECB4053L) and then incubated overnight at 4°C with primary antibodies. The membranes were probed with the following antibodies: anti-PNN (Novus Biologicals, NBP1-59136, Centennial, CO, USA) at 1 μg/mL dilution, anti-GAPDH (Merck, G8795) at 1:10,000 dilution and anti-β-Actin (Merck, A5441) at 1:4000 dilution. Bound primary antibodies were detected using HRP-conjugated secondary antibodies, mouse anti-rabbit (Merck, R3155) and rabbit anti-mouse (Merck, A9044) at a 1:10,000 dilution respectively and incubated 1 h at room temperature. After washing in TPBS, ECL (Bio-Rad, 1705061) was used to visualize the peroxidase-coated bands using the ChemiDoc MP Imaging System (Bio-Rad, Vers 3.0.1.14). Densitometric analysis was performed using Quantity One software (Bio-Rad, v4.6.6, PC). Western blot experiments were performed in three independent biological replicates.

### 2.6 Flow Cytometry Analysis (FACS) of Apoptosis

SW620 cells were seeded in six-well plates and subjected to the previously indicated treatments (*n* = 3 independent biological replicates). Following treatment, cells were detached using trypsin solution (Euroclone, ECM0920D), collected together with the corresponding supernatants, centrifuged, and resuspended in 500 μL of 1× Annexin Binding Buffer (BD Biosciences, 556454, Franklin Lakes, NJ, USA). Cells were subsequently stained with 1 μL of BV421-Annexin V (BD Biosciences, 563976, Franklin Lakes, NJ, USA) and 5 μL of 7-Amino-ActinomycinD (7-AAD) (BD Biosciences, 559925), followed by incubation for 30 min at room temperature in the dark. Samples were analyzed using a BD FACS Canto II flow cytometer (BD Biosciences, 338962), and data were processed with FACSDiva software (BD Biosciences, v9.0). At least 10,000 events were acquired for each sample. No fluorescence compensation was applied during data acquisition or analysis. Cell populations were first identified based on Forward Scatter Area (FSC-A) versus Side Scatter Area (SSC-A) to exclude debris. Doublets were excluded by sequential gating on FSC-H versus FSC-W and SSC-H versus SSC-W, selecting the singlet population. Viable and apoptotic cell populations were analyzed within the singlet gate using BV421-Annexin V and 7-AAD fluorescence. Quadrants were established based on unstained cells. Early apoptotic cells were defined as Annexin V-positive and 7-AAD-negative, whereas late apoptotic cells were both Annexin V- and 7-AAD-positive. Necrotic cells were identified as Annexin V-negative and 7-AAD-positive, while viable cells were double-negative for both markers.

### 2.7 Invasion, Migration and Clonogenic Assay

*Invasion*. Cell invasion was assessed with the modified Boyden chamber (48-multiwell plates; Neuroprobe, Gaithersburg, MD, USA) as previously reported with some modifications [11,12]. Briefly, Polyvinyl pyrrolidone-free polycarbonate filters, 8 μm pore size, were coated with Matrigel (0.5 mg/mL, Merck Merck-Millipore, CLS354230, Darmstadt, Germany), [O2] and incubated for 2 h at 37°C, 5% CO_2_, to allow the matrix to polymerize. Once polymerized, it was rehydrated with serum-free medium for 1 h at 37°C before its use. 20 × 10^3^ SW620 cells in DMEM (Euroclone, ECM0728L) with 1% FBS (Euroclone, ECS5000L) were loaded in the upper wells, while DMEM (Euroclone, ECM0728L) with 10% FBS was added to the lower wells, and incubated at 37°C for 48 h. Methanol-fixed cells were stained with Diff-Quik (Dade Behring, 130832, Dudingen, Switzerland), and cell invasion was measured by microscopic (NIKON Labophot, Minato-ku, Tokyo, Japan) evaluation by manual counting the number of cells moved across the filter in ten randomly selected fields at magnification 400×. Results are expressed as total counted cells/well. Each experimental point within a single experiment was measured in triplicate.

*Migration*. SW620 cells (1 × 10^5^) were cultured in 24-well plates and after they reached 95% confluence, a straight cell-free wound was made by manual scratching using a sterile 200 μL pipette tip [11,12]. Cells were washed with PBS twice to eliminate any cellular debris and a DMEM (Euroclone, ECM0728L) fresh medium was added during all the experiment, which served as the negative control (1% FBS, control time 0 h). After 48 h, cells migrated into the wounded area were visualized and photographed using an inverted light microscope (Nikon, Diaphot TMD, Nikon, Minato-ku, Tokyo, Japan) equipped with a digital acquisition system (Nikon Digital DS L-1, Nikon, Minato-ku, Tokyo, Japan). The area covered by cells was calculated with Fiji/ImageJ software (version 1.46, National Institutes of Health, Bethesda, MD, USA) software. For each experimental point, two different images were analyzed from time 0 to 48 h. Results are reported as percent of migration rate, calculated using the formula: Migration rate (%) = (Area time 0 h − Area time 48 h)/Area time 0 h × 100%.

*Clonogenic assays*. Briefly, SW620 cells were seeded in 6-well plates in (800 cells/well) in complete medium (DMEM [S.I.A.L. Srl, DMEM-HPXA] supplemented with 10% FBS [S.I.A.L. Srl, yourSIAL-FBS-SA, Rome, Italy]). Cells were daily observed, and DMEM (S.I.A.L. Srl, DMEM-HPXA) fresh medium was added every 3 days. Experiments were stopped on day 7. Cells were fixed with cold methanol for 30 min and then stained with DiffQuick (Dade Behring, 130832, Dudingen, Switzerland). Then colonies were manually counted by visual inspection using a magnifying lens.

### 2.8 Seahorse XF Metabolic Flux Analysis

*Mito Stress Assay.* Stable transfected shRNA-Pnn or shRNA-C SW620 cells were seeded in XFe96 cell culture plates (6 × 10^4^ cells per well; 12 technical replicates; 2 independent biological replicates). 18 h post-plating, the culture medium was replaced with 180 μL of XF DMEM medium (Agilent Technologies, 103575-100, Milan, Italy) supplemented with 25 mM glucose (Agilent Technologies, 103577-100), 2 mM glutamine (Agilent Technologies, 103579-100), and 1 mM sodium pyruvate (Agilent Technologies, 103578-100). Cells were incubated for 1 h at 37°C in a non-CO_2_ incubator before the analysis. The oxygen consumption rate (OCR), used as an indirect measurement of mitochondrial oxidative phosphorylation, was quantified using the Seahorse Extracellular Flux Analyzer (XFe96, Agilent Technologies, S7800BR). Extracellular acidification rate (ECAR), which is dependent on the mitochondrial-derived CO_2_ and on that of glycolysis, was also quantified. The addition of the ATP synthase inhibitor oligomycin (oligo, 1.5 μM; Merck, 75351), the proton uncoupler FCCP (2 μM; Merck, C2920), the respiratory complex I inhibitor rotenone (Rot, 1.5 μM; Merck, R8875), and the respiratory complex III inhibitor antimycin A (AA, 1.5 μM; Merck, A8674) was carried out using the manufacturer’s standard acquisition protocol without any modification of the default settings. Following calibration, cells were equilibrated for 12 min prior to measurement. Each measurement cycle consisted of a 3-min mixing phase, no waiting period, and a 3-min measurement phase. Three measurement cycles were acquired under basal conditions and after sequential injections of oligo (port A), FCCP (port B), and Rot plus AA (port C), respectively. To correct for non-cellular background signals, four cell-free wells were included in each assay plate. Background values were subtracted using the Wave software (Agilent Technologies, 2.6.4.24 version) and incorporated into the final calculations of OCR, ECAR, and glycolytic proton efflux rate (glycoPER), thereby ensuring accurate assessment of cellular metabolic activity. Protein quantification using BCA assay (Thermo Scientific, A55860) was used to normalize the results. Basal respiration is calculated as the last rate measurement before oligomycin injection minus the non-mitochondrial respiration rate. Maximal respiration is calculated as the maximum rate measurement after FCCP injection minus the non-mitochondrial respiration rate. Spare respiratory capacity is calculated as the maximal respiration minus basal respiration. ATP production is calculated as the decrease in OCR upon oligomycin injection and represents the portion of basal respiration used to drive ATP synthesis. Non-mitochondrial oxygen consumption respiration rate is calculated as the minimum rate measurement after Rot/AA injection.

*Glycolytic Rate Assay.* Stable transfected shRNA-Pnn or shRNA-C SW620 cells were seeded in XFe96 cell culture plates (6 × 10^4^ cells per well; 12 technical replicates; 2 independent biological replicates). 18 h post-plating, the culture medium was replaced with 180 μL of XF DMEM (Agilent Technologies, 103575-100) supplemented with 25 mM glucose (Agilent Technologies, 103577-100), 2 mM glutamine (Agilent Technologies, 103579-100), and 1 mM sodium pyruvate (Agilent Technologies, 103578-100). Cells were incubated for 1 h at 37°C in a non-CO_2_ incubator before the analysis and OCR and ECAR were quantified using the Seahorse Extracellular Flux Analyzer (XFe96; Agilent Technologies, S7800BR). The Glycolytic Rate Assay discriminates between ECAR that is dependent on mitochondrial-derived CO_2_ and that of glycolysis by concomitantly measuring the amount of OCR, thereby calculating the total proton efflux rate (PER). This analysis is performed by measuring in real-time ECAR and OCR after Rot/AA (1.5 μM; Merck, R8875) and 2-Deoxy-D-glucose (50 mM; Merck, G211230) administration at the indicated times, using the instrument’s default acquisition settings. After a 12-min equilibration period, each cycle consisted of a 3-min mixing phase followed by a 3-min measurement phase, with no waiting period. Three measurement cycles were recorded at baseline and following sequential injections of Rot plus AA (port A) and 2-DG (port B). Four cell-free wells were included in each plate for background correction, and the resulting values were subtracted using Wave software (Agilent Technologies, 2.6.4.24 version) and incorporated into the final OCR, ECAR, and glycoPER calculations. Protein quantification BCA assay (Thermo Scientific, A55860) was used to normalize the results. Inhibition of mitochondrial function by Rot/AA allows the calculation of mitochondrial-associated acidification. The subtraction of mitochondrial acidification (mitoPER) from the total PER results in the glycoPER and allows to calculate basal and compensatory glycolytic activity.

### 2.9 Nanopore RNA-Seq Library Construction and Sequencing

The total RNAs from SW620 cells treated with siRNA against *Pnn* or scrambled siRNA were transformed into cDNA for sequencing following the strand-switching protocol provided by Oxford Nanopore Technologies. Transcriptomic profiling was performed using *n* = 3 independent biological replicates for siRNA-Pnn-treated cells and *n* = 3 independent biological replicates for scrambled control cells. 500 ng of total RNA was used to prepare library using the cDNA-PCR Barcoding Kit V14 (SQK-PCB114.24) (Oxford Nanopore Technologies, Oxford OX4 4DQ, UK) following the protocol provided by the manufacturer (https://nanoporetech.com/document/pcr-cdna-sequencing-v14-barcoding-sqk-pcb114-24?device=PromethION). The RNA was used to synthesize cDNA via reverse transcription using a Maxima H Minus Reverse Transcriptase (200 U/μL) with 5x RT Buffer (ThermoFisher, cat # EP0752) as indicated by the protocol. The amplification of the cDNA was performed using PCR with barcoded primers provided in the kit. After selecting full-length transcripts by PCR, the resulting barcoded cDNA was purified using Agencourt AMPure XP beads (Beckman Coulter™ cat # A63881) and quantified using a Qubit dsDNA HS Assay Kit (Invitrogen-Thermo Fisher Scientific, USA, Q32851) and Qubit fluorometer (Invitrogen, Q33216, MA, USA). Furthermore, the barcoded cDNA was analyzed in an Agilent 2100 Bioanalyzer (Agilent Technologies) using Bioanalyzer High Sensitivity DNA Analysis to observe the average length of the fragments, as the samples were multiplexed and knowledge of the length and concentration was necessary to calculate the fmol per sample and then load a total of 50–70 fmol into the flow cell. The libraries were loaded onto R10.4.1 flow cells (FLO-PRO114M) and sequenced on a PromethION 2 Solo device (Oxford Nanopore Technologies, Pro-Seq002). Sequencing was performed for 72 h, and real-time base-calling was carried out using the MinKNOW software (Oxford Nanopore Technologies version **P2S_9172_v1**).

Data analysis was performed on pass reads with a quality (Q) score ≥ 9. Initial quality assessment was conducted using NanoPlot (v1.47.0, Table S1) to visualize median read length, median quality score, longest read length, total read count, and the distribution of read length and quality over sequencing runtime. Raw FASTQ files were further filtered using Fastcat by removing reads with a quality score < 10 and read length < 500 bp. Reads were subsequently oriented and barcode-trimmed using Pychopper prior to alignment. Processed reads were aligned to the human reference genome (GRCh38) using Minimap2. SAM files were converted to BAM format and sorted using SAMtools prior to gene quantification with featureCounts in long-read mode.

### 2.10 Bioinformatic Analysis

Pod5 files were base called using Dorado (v0.9.1) with the r10.4.1 super high accuracy model and aligned with minimap2 (v2.24-r1122) to the human genome reference cDNA (Homo_sapiens_assembly38.fasta from ensembl, https://www.ensembl.org/) using the standard parameter for nanopore sequencing (-ax map-ont). Read counts were estimated using feature Counts from the RSubread package (v 2.10.5) in R. Given the nature of the sequencing data, the software was configured to process single-end long reads without strand specificity. To ensure a comprehensive evaluation of the transcriptome, multi-mapping reads were included in the quantification, and reads overlapping with more than one genomic feature were allowed to be counted for all overlapping features without fractional assignment. Gene level annotation was conducted using the Homo sapiens GRCh38.113 annotation database (Homo_sapiens.GRCh38.113.chr.gtf). Differential gene expression analysis was performed using DESeq2 to identify differentially expressed genes (DEGs). No manual pre-filtering for low counts was performed on the raw count matrices before differential analysis. Instead, the independent filtering algorithm inherent to DESeq2 was utilized to dynamically remove lowly expressed features and maximize statistical power. The complete script and used parameters can be found in the (Tables S2 and S3). A sample information dataframe was constructed to define the experimental groups, assigning three replicates to the “Control” condition and three replicates to the “Test” condition. The generalized linear model was fitted using a design formula centered on the experimental condition (~condition), with the “Control” group serving as the reference baseline. Differential expression analysis was performed using the R package DESeq2 (v 1.36.0). Genes were considered significantly differentially expressed at an adjusted *p* value < 0.05, without applying a log2 fold change threshold. The ±0.58 log2 fold change cutoff was used only for descriptive categorization and downstream visualization analyses.

### 2.11 Gene Set Enrichment Analysis

Gene set enrichment analysis (GSEA) was performed using the fgsea package (v. 1.36.0) in R to identify perturbed biological pathways. Genes were ranked according to the Wald test statistic obtained from DESeq2 differential expression analysis. The ranked gene list was tested for enrichment against the curated gene set collections from Gene Ontology (GO) terms (c5.all.v2024.1.Hs.symbols.gmt). Enrichment was assessed using 10,000 permutations, and pathways with a Benjamini-Hochberg adjusted *p*-value < 0.05 were considered statistically significantly enriched.

### 2.12 Statistical Analysis

Statistical analyses were performed using unpaired two-tailed Student’s *t*-tests or Mann–Whitney tests, depending on data distribution. For comparisons involving more than two groups, two-way ANOVA followed by Tukey’s, Fisher or Sidak’s *post hoc* test was applied, *p* value < 0.05 was considered statistically significant. Unless otherwise specified, all experiments were performed with at least three independent biological replicates.

## 3 Results

### 3.1 Pnn Silencing Determines a Transcriptional Reprogramming of SW620 and HCT-116 Cells

To delineate the transcriptional consequences of *Pnn* silencing in an intrinsically resistant CRC cell model, we first transiently silenced *Pnn* in SW620 by specific siRNAs. RT-PCR and western blot analyses confirmed ~80% reduction in *Pnn* mRNA and protein levels compared with control cells (*p* ≤ 0.05, Fig. 1A). Successively, we performed RNA sequencing followed by differential gene expression and gene set enrichment analyses. Across an average of 14,633 expressed genes, 1494 genes were identified as significantly modulated between Pnn-silenced and control cells (at least an absolute 1.5-fold change in expression baseline and an adjusted *p*-value less than 0.05). Among these, 797 genes were upregulated, and 697 were downregulated, indicating that *Pnn* loss triggers a balanced yet extensive remodeling of the cellular transcriptome. To obtain a functional overview of these changes, we conducted a Gene Set Enrichment Analysis (GSEA) using Gene Ontology Biological Process (GOBP) gene sets. A total of 1299 pathways reached statistical significance (padj < 0.05), reflecting both the depth of transcriptional perturbation and the inherent redundancy within GO annotations. To identify the most biologically relevant processes, all significant pathways were ranked by their Normalized Enrichment Score (NES), and the top 100 upregulated and top 100 downregulated pathways were selected for focused evaluation. These were further categorized into seven major functional groups encompassing cell cycle and proliferation (*n* = 8), genomic instability (*n* = 5), DNA damage response and repair (*n* = 3), energy metabolism (*n* = 5), p53 and apoptosis signaling (*n* = 6), oncogenic signaling (*n* = 4), and autophagy and protein degradation (*n* = 3). For each pathway, the two most differentially expressed genes concordant with the pathway direction were identified (Fig. 1B). *Pnn* silencing led to a pronounced promotion of proliferative programs, with cell cycle-related pathways being among the most significantly enhanced (mean NES = 1.90; range: from 1.64 to 2.24). Processes such as mitotic nuclear division (NES = 2.24, padj = 8.49 × 10^−9^) and cell division (NES = 2.19, padj = 1.10 × 10^−12^) exhibited the strongest upregulation, suggesting that *Pnn* is required to repress transcriptional programs that drive mitotic progression and cell cycle continuity. In agreement, pathways linked to genomic integrity and chromosome segregation were markedly upregulated (mean NES = 1.98), particularly sister chromatid segregation (NES = 2.19, padj = 1.29 × 10^−7^) and spindle-kinetochore attachment (NES = 2.00, padj = 2.54 × 10^−3^). Further inspection revealed that DNA damage response and repair mechanisms were also significantly promoted (mean NES = 1.41), including double-strand break repair regulation (NES = 1.66, padj = 0.025) and DNA replication (NES = 1.61, padj = 0.025). The concurrent enhancement of replication and repair programs implies that PNN may interfere with genomic maintenance. Consistent with this pattern, oncogenic survival and signaling cascades exhibited coherent upregulation (mean NES = 1.24), with the MAPK signaling cascade being among the most significantly affected (NES = 1.61, padj = 2.70 × 10^−3^). Additionally, autophagy and protein degradation pathways were globally upregulated (mean NES = 1.13), including macroautophagy (NES = 1.56, padj = 8.00 × 10^−3^). In contrast to the broad repression of proliferative and survival pathways, energy metabolism showed a robust downregulation (mean NES = −1.82; range: 1.13–2.35). Key pathways related to oxidative phosphorylation (OXPHOS) (NES = −2.35, padj = 5.96 × 10^−7^) and the electron transport chain (NES = −2.31, padj = 8.56 × 10^−7^) were significantly enriched, indicating a metabolic impairment of mitochondrial respiration. This metabolic reprogramming prevents a compensatory adaptation to sustain ATP production under promoted proliferative demand or altered biosynthetic requirements. Interestingly, p53 and apoptosis-related pathways displayed a mixed regulatory pattern (mean NES = 0.47; range: −2.11 to 1.13). Notably, p53-mediated signal transduction was significantly downregulated (NES = −2.11, padj = 7.02 × 10^−3^). The suppression of p53 signaling underscores a shift to a proliferative uncontrolled transcriptional state. Collectively, these results define a comprehensive transcriptional reprogramming triggered by *Pnn* silencing, encompassing upregulation of cell cycle and genome integrity pathways, promotion of oncogenic and pro-survival signaling, increased autophagic and proteins homeostasis-related activity, reduced mitochondrial oxidative metabolism, and inhibition of p53-dependent stress signaling. This integrated molecular profile suggests that *Pnn* depletion not only induces proliferative potential but also compromises DNA repair and adaptive responses by impairment of the Positive Regulation of Apoptotic Signaling Pathway and pathways related to energy metabolism, thereby potentially increasing cellular susceptibility to DNA-damaging and metabolic stress due to insufficient metabolic pathways sustaining this increased proliferation. To increase the robustness of the observed transcriptional and functional effects and to enable the investigation of long-term phenotypic consequences of *Pnn* depletion, we generated stable *Pnn*-silenced SW620 cells using a lentiviral shRNA approach. As shown in Fig. 1C, both *Pnn* mRNA and protein levels were substantially reduced (~76% and ~65% decrease relative to mRNA and protein, respectively) in shRNA-Pnn relative to shRNA control cells. Notably, *Pnn* stable knockdown recapitulated the transcriptional changes observed upon transient silencing, confirming the consistency of *Pnn*-dependent gene regulation. As shown in Fig. 1D, both siRNA- and shRNA-*Pnn*-silenced cells exhibit the same deregulation profile.

To extend our findings to another colon cancer cell line widely used in chemoresistance studies, *Pnn* silencing was performed in HCT-116 cells by a lentiviral shRNA approach. As shown in Fig. 2A, *Pnn* silencing resulted in a marked reduction of *Pnn* mRNA and protein expression (~68% and ~78% decrease relative to mRNA and protein, respectively). Moreover, the analysis of the same gene panels previously evaluated in SW620 cells demonstrated that *Pnn* silencing affects expression of key genes involved in previously described cellular pathways also in HCT-116 cells (Fig. 2B), further supporting the involvement of *Pnn* in the regulation of molecular networks associated with colon cancer progression and drug resistance.

**Figure 1:**
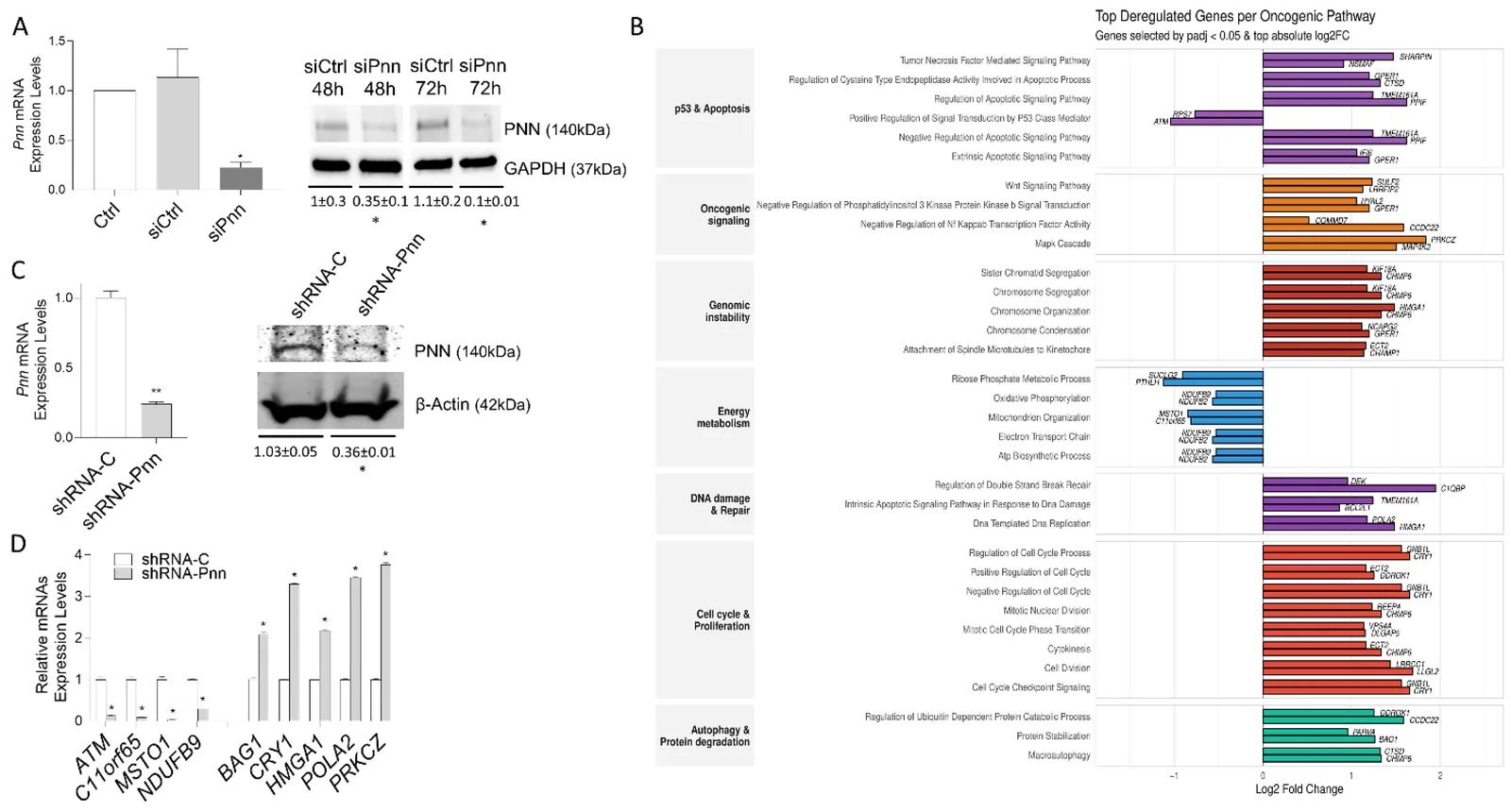
***Pnn* silencing drives transcriptional reprogramming in SW620 cells.** (**A**) shows the *Pnn* silencing in SW620 cells by means of siRNAs at 48 h and 72 h, confirmed by RT-qPCR and western blot (~80% reduction vs. siCtrl). (**B**) Bar plot showing the two most deregulated genes in each oncogenic pathway identified by RNA-seq analysis (padj < 0.05), indicating broad transcriptomic remodeling. GSEA showed enrichment of pathways related to cell cycle and proliferation, genomic stability and DNA repair, oncogenic signaling, and autophagy, while energy metabolism (e.g., OXPHOS) and p53 signaling were downregulated. (**C**) shows the effects of *Pnn* stable silencing (shRNA-Pnn) in SW620 cells, confirmed by RT-qPCR and western blot. (**D**) Validation of selected differentially expressed genes by RT-PCR analysis in shRNA-C and shRNA-Pnn stable-silenced cells. Gene expression changes are consistent with RNA-seq results. Data are presented as mean ± SD. *p < 0.05; **p < 0.001.

**Figure 2:**
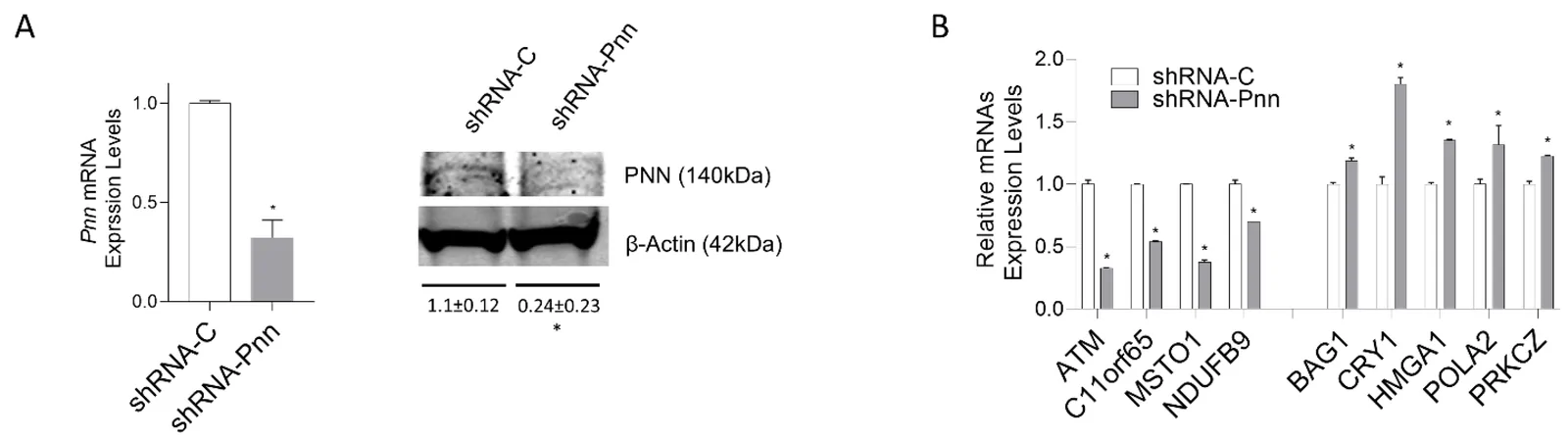
***Pnn* silencing affects the expression of selected genes in HCT-116 cells.** (**A**) shows the effects of *Pnn* stable silencing (shRNA-Pnn) in HCT-116 cells, as confirmed by RT-qPCR (~70% reduction vs. shRNA-C control). (**B**) Validation of selected differentially expressed genes by RT-qPCR analysis in shRNA-C and shRNA-Pnn HCT-116 stable-silenced cells. Data are presented as mean ± SD. *p < 0.05.

### 3.2 Pnn Silencing Reduces Clonogenicity, Motility and Invasion of SW620 Cells

We evaluated the role of *Pnn* in modulating the clonogenic potential and invasive capacity of stable shRNA-Pnn SW620 cells after 1 week after plating for clonogenic potential and 48 h after plating for cell mobility and invasion. As shown in Fig. 3A, stable silencing of *Pnn* markedly reduced the clonogenic ability of the cells compared to the control. Representative images (Fig. 3B) further illustrate a clear decrease in cell invasiveness following *Pnn* knockdown. To further support these findings, we also performed a wound-healing assay, revealing that *Pnn* knockdown affects motility of SW620 cells (Fig. 3C). Consistently, quantitative analysis revealed a significant reduction in colony formation, number of invading cells and migration rate in shRNA-Pnn samples compared with the control (*p* < 0.004). These results suggest that *Pnn* contributes to both clonogenic growth and the invasive phenotype of these cells, and that its depletion significantly impairs their proliferative and invasive potential.

**Figure 3:**
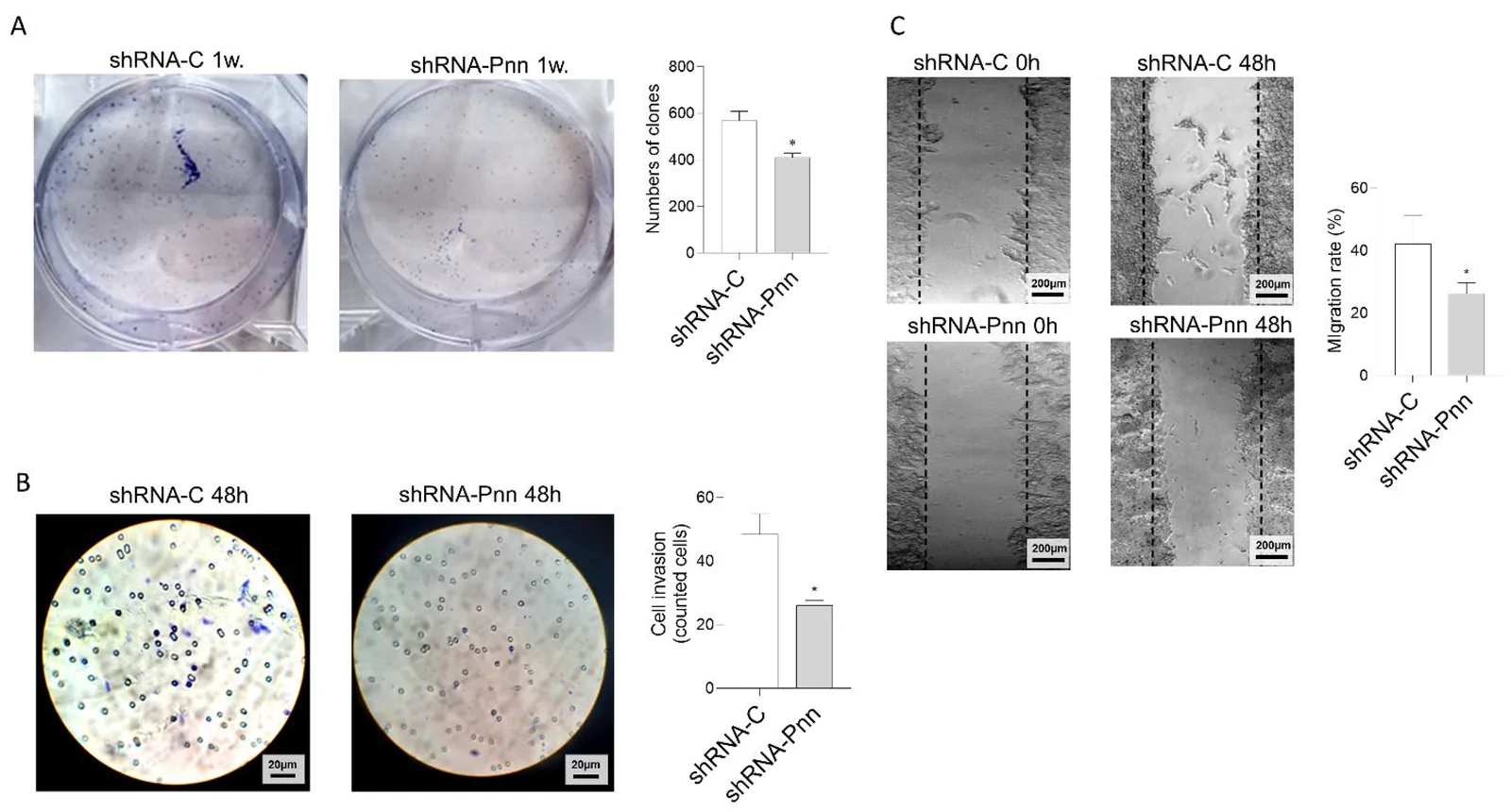
***Pnn* silencing reduces clonogenicity and invasion of SW620 cells.** (**A**) Clonogenic assay showing a significant reduction in colony formation in *Pnn*-silenced (shRNA-Pnn) cells compared with control (shRNA-C) at 1 week. (**B**) Representative images of invasion assays demonstrating decreased invasive capacity upon *Pnn* silencing. Scale bar: 20 μm. (**C**) Wound-healing assay showing impaired migratory ability in *Pnn* silenced cells compared to control at 48 h. Scale bar: 200 μm. All images shown are representative of four independent experiments. Quantification of four independent experiments confirms a significant reduction in both clonogenic growth and invasive potential following *Pnn* depletion. Data are presented as the mean ± SD. *p < 0.05.

### 3.3 Pnn Silencing Impairs Mitochondrial Respiration without Inducing Glycolytic Compensation in SW620 Cells

Based on transcriptomic data, we performed experiments to evaluate the impact of *Pnn* silencing on the metabolic activity of SW620 cells, by Seahorse analyses (Fig. 4). In the Mito Stress Test, the bioenergetic profile indicates a marked impairment of mitochondrial function in *Pnn*-silenced cells compared with control. Parameters derived from the oxygen consumption rate (OCR) measurements, reflecting mitochondrial respiration and oxidative phosphorylation activity, were overall reduced upon *Pnn* knockdown (Fig. 4A,B). Basal respiration was lower in shRNA-Pnn cells, suggesting either a diminished energetic demand sustained by oxidative phosphorylation or an intrinsic defect in electron transport chain activity. Furthermore, maximal respiration, assessed following the addition of the uncoupling agent FCCP, was also attenuated in shRNA-Pnn cells, indicating a limited ability to increase respiratory flux upon pharmacologically induced mitochondrial uncoupling during the Seahorse Mito Stress assay. Consistently, the spare respiratory capacity was reduced, suggesting a reduced mitochondrial respiratory adaptability and a compromised ability to respond to increased energetic demand during the assay conditions. In contrast, proton leak, ATP-linked respiration, and non-mitochondrial oxygen consumption did not differ significantly in *Pnn*-silenced cells compared with control, suggesting that the primary effect of *Pnn* silencing specifically impacts mitochondrial bioenergetic performance rather than non-mitochondrial oxygen-consuming processes. In the Glyco Rate test, proton efflux rate (PER) measurements indicated that basal glycolysis, basal proton efflux rate, and compensatory glycolysis were comparable between shRNA-Pnn and control cells, with no statistically significant differences observed (Fig. 4C, D). The dynamic response following rotenone/antimycin A injection, which shifts ATP production toward glycolysis, showed similar kinetics and amplitude in both groups. Moreover, inhibition with 2-deoxyglucose reduced glycolytic activity to comparable extents, confirming that the glycolytic machinery remains functionally intact in *Pnn*-silenced cells. Collectively, these data indicate that, in agreement with results obtained by transcriptomic analysis, *Pnn* silencing selectively impairs mitochondrial oxidative phosphorylation in SW620 cells without triggering a compensatory increase in glycolytic flux. The concomitant reduction in basal respiration, maximal respiration, and spare respiratory capacity points to a diminished mitochondrial fitness. Importantly, basal glycolytic activity and compensatory glycolysis, assessed by glycoPER measurements following rotenone/antimycin A treatment, were not significantly different between shRNA-Pnn and control cells. Therefore, *Pnn*-deficient cells did not exhibit a detectable glycolytic compensatory response under the experimental conditions tested. These findings support the presence of impaired mitochondrial respiratory performance; however, additional mechanistic studies will be required to determine whether this phenotype reflects a direct role of PNN in metabolic regulation or a secondary consequence of broader transcriptional and cellular alterations induced by *Pnn* depletion.

**Figure 4:**
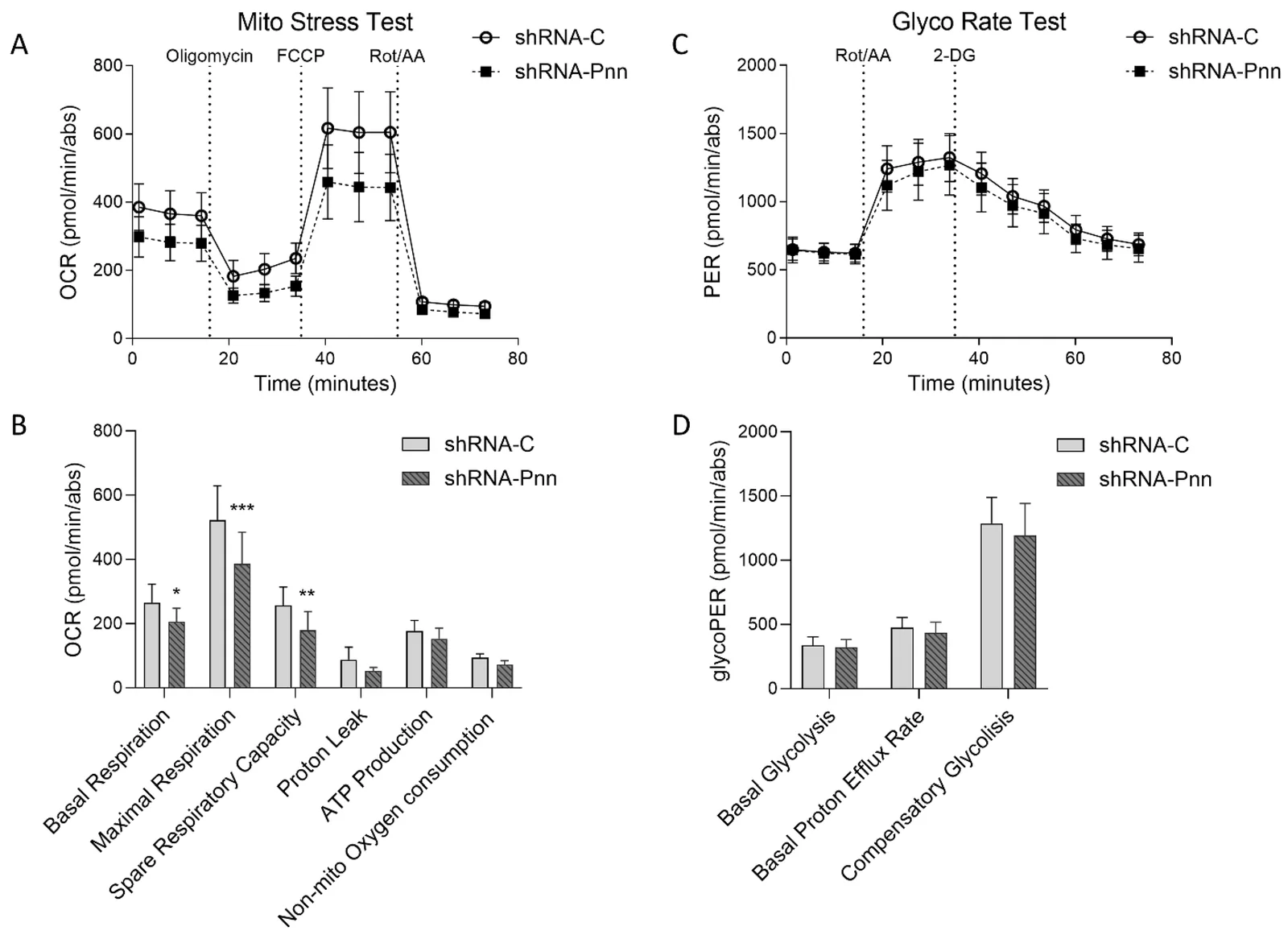
***Pnn* silencing promotes metabolic reprogramming.** (**A**) Seahorse XFe96 Mito Stress Test on shRNA-C and shRNA-Pnn SW620 cells. OCR was calculated in real-time after administration of ATP synthase inhibitor oligomycin, FCCP, and respiratory complex I inhibitor Rot together with respiratory complex III inhibitor AA (Rot/AA). (**B**) Basal and maximal respiration, spare respiratory capacity, proton leak, ATP production and non-mitochondrial oxygen consumption were calculated as described in the Materials and Methods section and normalized on protein content. Data are represented as mean ± SD. Two-way ANOVA followed by Fishers’s LSD test. *p < 0.05, **p < 0.01, ***p < 0.001. (**C**) Seahorse XFe96 Glycolysis Stress Test was performed; PER was calculated in real-time after the administration of Rot/AA and 2-deoxyglucose (2-DG). (**D**) Basal glycolysis, basal proton efflux rate and compensatory glycolysis were calculated as described in the Materials and Methods section and normalized on protein content. Data are represented as mean ± SD. Two-way ANOVA followed by Fishers’s LSD test.

### 3.4 Pnn Silencing Affects Chemotherapy-Induced Cytotoxicity in SW620 and HCT-116 Cells

To assess baseline drug sensitivity of SW620 cells to the OHP and 5-FU chemotherapeutics, IC_50_ values were measured after 72 h of treatment, revealing an IC_50_ of 3.453 ± 2.16 μM for OHP and an IC_50_ of 21.41 ± 5.81 μM for 5-FU (Fig. 5A). The difference between the two IC_50_ values was significant (*p* ≤ 0.005), confirming OHP’s higher cytotoxic efficacy. To determine whether *Pnn* silencing modulates the cellular response to chemotherapeutic agents, we next assessed the cytotoxic effects of OHP and 5-FU exposure in transiently *Pnn*-silenced or control SW620 cells. Cells were incubated with increasing concentrations of either drug for 72 h, and cell viability was evaluated. Fig. 5B shows a significant reduction of viability in transiently *Pnn*-silenced cells compared to control, particularly at higher drug concentrations. At IC_50_ doses, *Pnn*-silenced cells showed about 45% decrease in viability compared with control cells upon exposure to OHP (*p* < 0.05, Fig. 5B, left) and about 40% decrease upon exposure to 5-FU (*p* < 0.05, Fig. 5B, right). Supplementary Table S2 summarized the values obtained by MTT assay. This enhanced cytotoxicity was already appreciable at lower doses, suggesting that the loss of *Pnn* lowers the apoptotic threshold and sensitizes cells to chemotherapeutic stress in a broad, rather than strictly dose-restricted, manner. To address the role of stable *Pnn* knockdown in chemoresistance of SW620 cells, similar experiments were performed on shRNA-Pnn or shRNA-C cells. Basal MTT assays revealed no significant differences in viability between shRNA-Pnn and shRNA-C cells, indicating that lentiviral transduction, selection and *Pnn* silencing did not affect viability (Fig. 5C). *Pnn* stable knockdown markedly increased drug-induced cytotoxicity, reducing cell viability by ~45% after OHP exposure and ~40% after 5-FU compared with shRNA-C controls (*p* < 0.001) at IC_50_ doses. Consistently, IC_50_ values were significantly decreased in shRNA-Pnn cells, showing a ~3.5-fold reduction for OHP (3.8 ± 0.4 μM vs. 1.1 ± 0.2 μM) and a ~3.1-fold reduction for 5-FU (15.5 ± 1.2 μM vs. 5.0 ± 0.8 μM) (Fig. 5C left and right, respectively; Supplementary Table S3 summarizes the values obtained by MTT assay). Fig. 6 shows the FACS analysis employed to evaluate apoptosis contribution in the observed viability reduction. Annexin V/7-AAD FACS analysis performed after 72 h exposure to OHP or 5-FU at IC50 concentrations demonstrated a pronounced increase in both early and late apoptotic populations, together with an increase in necrotic cells, in *Pnn*-stably silenced cells compared with controls, in line with the reduction in cell viability. A similar analysis was conducted in stably *Pnn*-silenced HCT-116 cells. Baseline drug sensitivity was measured after 72 h of treatment with OHP and 5-FU, revealing an IC_50_ of 1.1 ± 0.29 μM for OHP and an IC_50_ of 10.86 ± 1.22 μM for 5-FU (Fig. 7A). *Pnn* stable knockdown markedly increased drug-induced cytotoxicity also in HCT-116, reducing cell viability by ~32% after OHP exposure and ~31% after 5-FU compared with shRNA-C controls (*p* < 0.005) at IC_50_ doses. Consistently, IC_50_ values were significantly decreased in shRNA-Pnn cells, showing a ~2.4-fold reduction for OHP (0.491 ± 0.093 μM vs. 0.2019 ± 0.097 μM) and a ~2.2-fold reduction for 5-FU (2.44 ± 0.47 μM vs. 1.10 ± 0.29 μM) compared to shRNA-C cells (Fig. 7B; Table S4 summarized the values obtained by MTT assay).

Together, these data demonstrate that both transient and stable *Pnn* silencing strongly sensitizes SW620 and HCT-116 colorectal cancer cells to OHP- and 5-FU-induced cytotoxicity. *Pnn* depletion consistently enhances apoptotic responses and significantly lowers drug IC_50_ values, supporting a key role for *Pnn* in sustaining chemoresistance.

**Figure 5:**
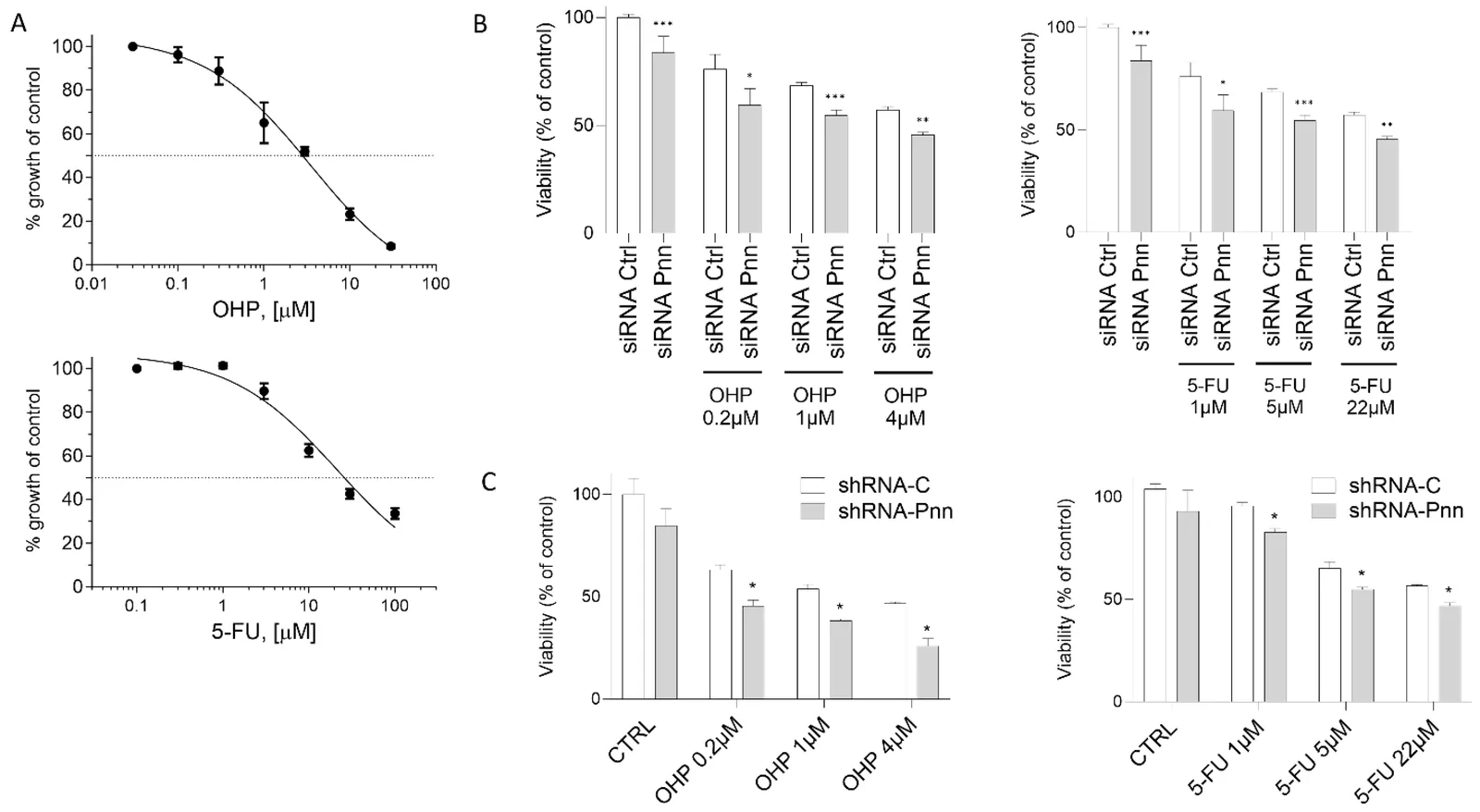
***Pnn* silencing affects chemotherapy-induced cytotoxicity in SW620 cells.** (**A**) Identification of SW620 cells baseline drug sensitivity to the OHP and 5-FU. (**B**) Comparative cytotoxicity of OHP and 5-FU (OHP 0.2, 1, 4 μM or 5-FU 1, 5, 22 μM) after 72 h in transient SW620 siRNA-Pnn and siRNA-Ctrl cells. (**C**) Comparative cytotoxicity of OHP and 5-FU (OHP 0.2, 1, 4 μM or 5-FU 1, 5, 22 μM) in stable SW620 shRNA-Pnn and shRNA-C Ctrl cells. Data represent the mean ± SD. Two-way ANOVA followed by Sidak’s multiple comparison test. *p < 0.05, **p < 0.01, ***p < 0.001.

**Figure 6:**
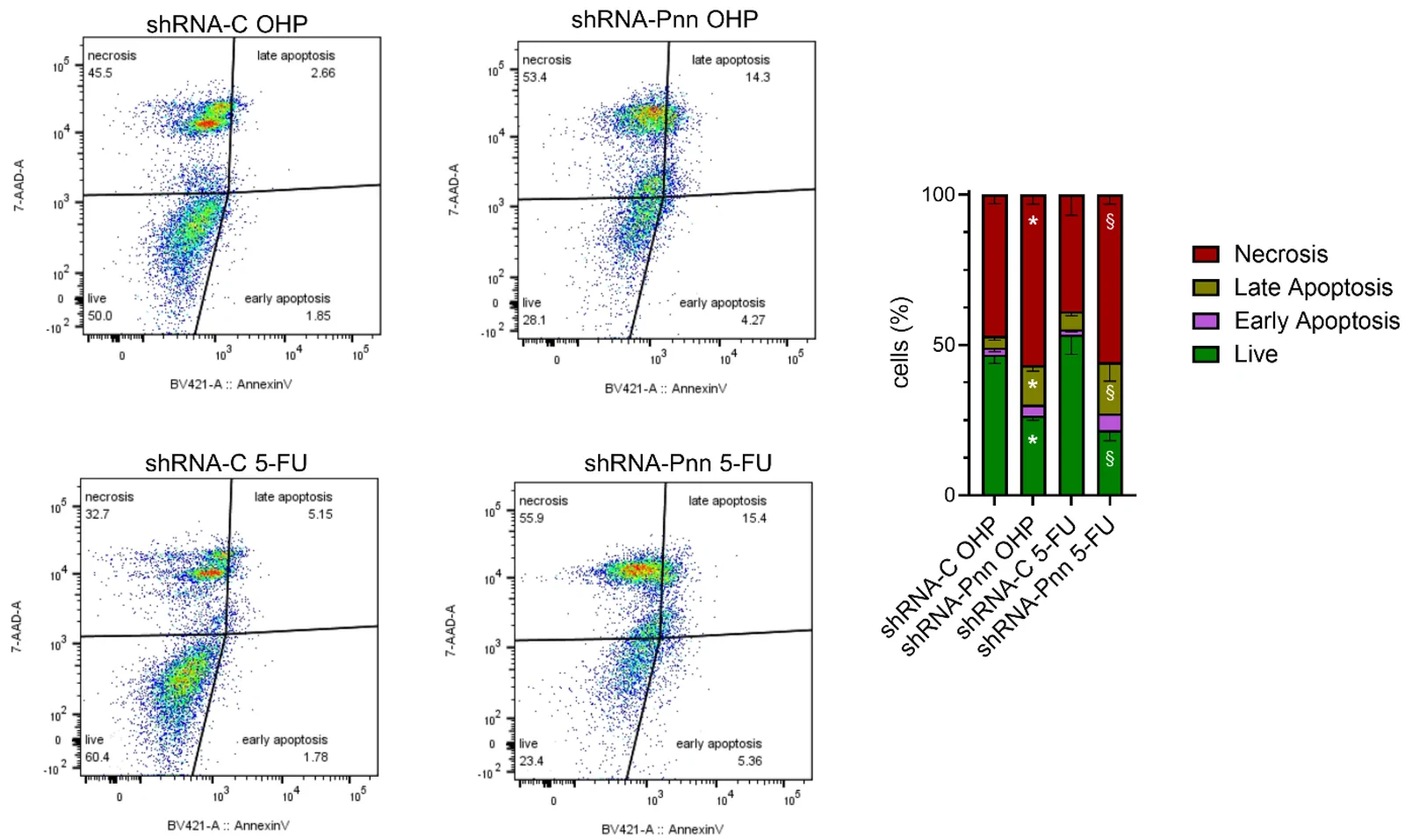
***Pnn* silencing increases apoptosis of SW620 cells treated with OHP and 5-FU.** Apoptosis and necrosis were assessed by flow cytometry using Annexin V/7-AAD staining. (**Left**) Representative Annexin V/7-AAD dot plot of shRNA-C and shRNA-Pnn SW620 cells treated with OHP and 5-FU. (**Right**) Quantitative analysis shows the percentage of live, early apoptotic, late apoptotic, and necrotic cells. Data are represented as mean ± SD. Two-way ANOVA followed by Tukey’s *post hoc* test. *p < 0.05 vs. shRNA-C OHP; ^§^*p* < 0.05 vs. shRNA-C 5-FU.

**Figure 7:**
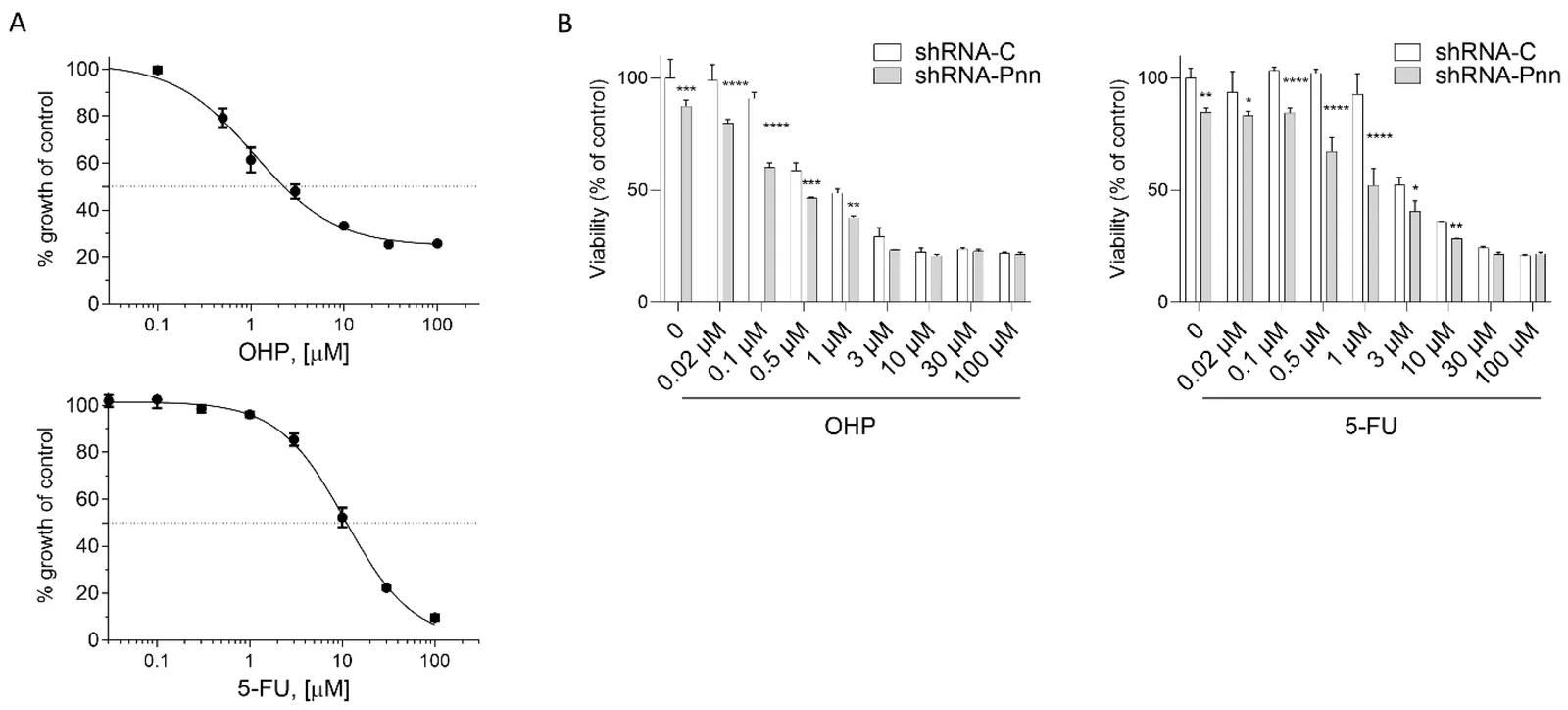
***Pnn* silencing affects chemotherapy-induced cytotoxicity in HCT116 cells.** (**A**) Identification of HCT-116 cells baseline drug sensitivity to the OHP and 5-FU. (**B**) Comparative cytotoxicity of OHP and 5-FU (0, 0.02, 0.1, 0.5, 1, 3, 10, 30, 100 μM) in stable HCT-116 shRNA-Pnn and shRNA-C Ctrl cells. Data represent the mean ± SD. Two-way ANOVA followed by Sidak’s multiple comparison test. *p < 0.05, **p < 0.01, ***p < 0.001, ****p < 0.0001.

## 4 Discussion

In this study, we identify PNN, a spliceosome and transcriptional regulatory protein, associated with CRC clonogenic and invasive potential, bioenergetic regulation, and chemoresistance, integrating molecular, cellular, and transcriptomic evidence to reveal how its depletion fundamentally rewires survival programs under therapeutic stress. Previous correlative analyses highlighted an association between elevated PNN expression and poor disease outcome [9,10], but mechanistic understanding remained limited. Here, our data suggest that PNN may contribute to RNA processing, cell-cycle regulation, stress signaling, and metabolic homeostasis, processes potentially involved in treatment tolerance. Our functional assays reveal that both transient and stable *Pnn* knockdown significantly enhance sensitivity to OHP and 5-FU in an *in-vitro* model of CRC, of current therapeutic regimens [13]. This chemo-sensitization aligns with emerging evidence suggesting that RNA-processing regulators act as pivotal modulators of the cancer stress phenotype [14]. RNA-binding proteins and spliceosome components have been shown to maintain proper expression of DNA repair machinery, apoptotic regulators, and metabolic enzymes [15]. Therefore, the increased apoptotic events observed following PNN deregulation may reflect a cumulative disruption of multiple pro-survival networks. These results also align with the concept of therapy-induced adaptation, in which tumor cells reorganize transcriptional and post-transcriptional programs to survive genotoxic stress [16]. PNN may participate in adaptive cellular responses, as suggested by the transcriptional remodeling observed following its depletion. RNA-seq and GSEA analyses reveal a significant upregulation of pathways associated with cell-cycle progression, mitotic division, and chromosomal segregation following *Pnn* depletion, suggesting that PNN normally contributes to restraining proliferative transcriptional programs. Aberrant activation of mitotic programs in the context of impaired metabolic and stress adaptation pathways may promote replication stress and genomic instability, conditions known to increase susceptibility to chemotherapeutic agents. Aberrant mitotic checkpoint activity, weakened kinetochore dynamics, and compromised replication fork stability are widely acknowledged contributors to chemosensitivity in CRC [17]. Thus, deregulation of these pathways could contribute to increased vulnerability to DNA-damaging agents, although additional studies are required to establish causal relationships. Furthermore, transcriptional signatures associated with DNA replication and repair processes were also upregulated upon *Pnn* silencing, suggesting an adaptive attempt of the cells to maintain genome integrity under conditions of proliferative pressure. However, such compensatory activation may not be sufficient to preserve genomic stability, particularly in the presence of chemotherapeutic stress. Loss of replication competence and increased replication stress make cells more vulnerable to DNA-damaging agents like OHP, which induces intra- and inter-strand crosslinks [18], and 5-FU, which results in misincorporation and replication stalling [19]. The involvement of PNN in splicing regulation represents a compelling mechanistic axis linking its loss to extensive transcriptomic perturbations. Alternative splicing influences multiple cancer hallmarks, including escape from apoptosis, invasive behavior, and drug resistance [20]. Several major splicing regulators, including SRSF proteins, hnRNP families, and RBFOX members, have been linked to CRC progression and treatment response [21]. Our findings support a possible involvement of PNN among factors associated with a resistant phenotype by determining isoform output of crucial stress-response genes. Although our analysis focused on gene-level transcriptional changes, the magnitude of the observed shifts suggests that PNN may influence splicing events involved in DNA repair, mitotic checkpoint regulation, metabolism, and apoptotic signaling [22]. Future systematic profiling of full-length transcripts and splice junctions will be essential to map PNN-dependent splicing signatures, as recommended by recent multi-omics investigations in therapy-resistant tumors [23]. Another central observation emerging from our transcriptomic analysis is the upregulation of MAPK signaling-related gene signatures in *Pnn*-depleted cells. MAPK signaling is a well-established driver of CRC progression and acts as a mediator of resistance to standard therapies [24]. ERK signaling promotes cell-cycle progression, enhances DNA repair capacity, and establishes a pro-survival transcriptional state [25]. The paradoxical upregulation of MAPK-associated transcriptional programs observed here may reflect a compensatory attempt of the cells to sustain survival signaling following *Pnn* depletion, although the metabolic and stress-related impairments observed in these cells likely limit the effective execution of these pathways. Prior studies have linked PNN to activation of EGFR/ERK and PI3K/AKT pathways [26], further supporting the idea that PNN strengthens signaling circuits associated with therapeutic tolerance. Chemoresistant CRC cells typically exhibit profound metabolic plasticity, favoring the Warburg effect (i.e., aerobic glycolysis and increased lactate production), and the enhancement of antioxidant defenses [27]. Our transcriptomic and metabolic analyses consistently indicate a reduction in oxidative phosphorylation following *Pnn* depletion. Seahorse analyses confirmed a significant impairment of mitochondrial respiration, including reductions in basal respiration, maximal respiratory capacity, and spare respiratory capacity. Importantly, these analyses were performed under basal culture conditions. Notably, mitochondrial impairment was not accompanied by a detectable compensatory increase in glycolytic flux under the experimental condition tested. Furthermore, *Pnn* loss is associated with altered energy metabolism and increased apoptotic responses following chemotherapy exposure. While these findings suggest altered mitochondrial bioenergetic performance in *Pnn*-depleted cells, they do not demonstrate a direct transcriptional regulation of metabolic genes by PNN. Moreover, the observed metabolic phenotype may also reflect broader cellular consequences of Pnn depletion, including deregulated proliferative signaling, replication stress, or altered stress-response pathways. Therefore, additional mechanistic studies will be necessary to clarify whether PNN directly regulates mitochondrial metabolic programs or indirectly affects cellular bioenergetics through global transcriptional reprogramming. Although basal transcriptional signatures associated with p53 signaling appear reduced in *Pnn*-depleted cells, the marked increase in apoptosis observed after chemotherapeutic treatment indicates that *Pnn* loss lowers the threshold for stress-induced cell death. This suggests that PNN may regulate additional pathways controlling apoptotic susceptibility independently of canonical p53 transcriptional activation. Increased apoptotic sensitivity may therefore arise from the combined effects of metabolic dysfunction, replication stress, and impaired stress adaptation mechanisms. In addition, our transcriptomic analysis revealed an increase in autophagy-related transcriptional signatures following *Pnn* silencing, possibly reflecting an adaptive attempt to manage cellular stress and maintain protein homeostasis. However, despite this apparent activation of autophagy related pathways, the strong apoptotic response observed upon chemotherapy exposure suggests that these compensatory mechanisms are insufficient to restore cellular homeostasis under genotoxic stress. Furthermore, invasion assays revealed that *Pnn* deregulation significantly reduces CRC cell motility and invasiveness. These findings suggest an association between *Pnn* expression and migratory or invasive properties in CRC cells. The findings presented here have significant clinical relevance. Previous correlative studies suggested a possible association between *Pnn* expression and chemotherapy response [9,10]. Our findings support further investigation of *Pnn* as a potential therapeutic target. Pharmacological modulation of splicing regulators has gained traction, with early-stage inhibitors showing efficacy in hematologic and solid tumors. Targeting PNN-dependent pathways, especially those involved in RNA processing, metabolic homeostasis, and survival signaling, may represent a strategy to resensitize resistant CRC to conventional regimens. Despite the depth of the findings, some limitations must be acknowledged. Our model system, while highly informative, does not account for microenvironmental influences such as stromal interactions, hypoxia, and immune modulation that deeply affect chemotherapy response [28,29]. Future studies using patient-derived organoids, xenografts, or co-culture systems incorporating fibroblasts and immune cells will help validate the broader physiological relevance of PNN-dependent pathways. Although the drug concentrations used in this study fall within ranges commonly adopted in preclinical CRC *in vitro* models, further investigations using physiologically informed exposure systems and *in vivo models* will be required to better define the translational relevance of PNN-dependent chemosensitization. An additional limitation of the present study is that rescue/complementation experiments, which are generally considered the gold standard for confirming target specificity following gene silencing, were not performed. Therefore, although the phenotypic and transcriptomic changes observed after *Pnn* depletion are consistent with a functional role of PNN in chemoresistance, clonogenicity, invasion, and metabolic regulation, we cannot completely exclude the contribution of potential off-target effects associated with the silencing approaches used. To address this limitation, future studies will employ CRISPR/Cas9-mediated generation of *Pnn* knockout CRC cell lines followed by rescue experiments based on *Pnn* re-expression. Restoration of *Pnn* expression in the knockout background will allow us to determine whether the resistant phenotype, together with the associated molecular, metabolic, and functional parameters, can be recovered. These experiments will provide a more definitive validation of the specificity of PNN-dependent effects and further clarify its mechanistic contribution to therapy resistance. Additionally, our current RNA-seq analysis provides valuable pathway-level insights but cannot resolve isoform-level regulation. Advanced long-read sequencing approaches or single-cell multi-omics will be needed to dissect the full spectrum of PNN regulatory functions. Furthermore, a limitation of this study is that the Seahorse metabolic analyses were performed in only two independent experiments; therefore, these findings require further validation through higher-resolution, targeted metabolic profiling to comprehensively characterize the metabolic alterations induced by *Pnn* silencing. This study identifies PNN as a critical regulator of colorectal cancer progression, particularly affecting clonogenic and invasive potential, metabolic flexibility, and, notably, chemoresistance. Through complementary transient and stable loss-of-function approaches, we demonstrate that Pnn silencing profoundly restores sensitivity to OHP and 5-FU, enhancing apoptotic responses and reducing the cytotoxic threshold of both chemotherapeutic drugs. These functional effects are supported by extensive transcriptional reorganization, characterized by deregulation of mitotic and genome maintenance programs, impairment of mitochondrial oxidative metabolism, and altered stress-response signaling. Together, these findings suggest that PNN depletion is associated with transcriptional, metabolic, and phenotypic alterations that may contribute to chemotherapy sensitivity. PNN depletion was accompanied by reduced mitochondrial respiration, increased apoptotic responses, and enhanced chemosensitivity, although the mechanistic relationships among these processes remain to be fully elucidated.

## 5 Conclusion

This study demonstrates that PNN plays a central role in colorectal cancer cell adaptation to chemotherapy. PNN depletion sensitizes CRC cells to OHP and 5-FU while impairing mitochondrial function and reducing invasive potential, supporting its involvement in mechanisms underlying therapy resistance. Although additional mechanistic and *in vivo* studies are required, these findings identify PNN as a promising biomarker and potential therapeutic target for improving treatment response in colorectal cancer.

## Data Availability

RNA-seq data are deposited at NCBI BioProject data repository PRJNA1445922.

## Supplementary Materials

The supplementary material is available online at https://www.techscience.com/doi/10.32604/or.2026.084313/s1.
