# Supplementary material for: Pinin Modulation Restores Chemosensitivity in SW620 and HCT-116 Colon Cancer Cells
Source: Oncol Res. 2026 Oct 28;34(11):1. doi: 10.32604/or.2026.084313 (PMC13732370; doi:10.32604/or.2026.084313)
Supplement: Supplementary file 1 [file OncolRes-34-84313-s001.zip › Table_S4.docx]

**Table S4.**

Inhibitory effects of stable shRNA-Pnn respect to shRNA-C HCT116 cells line on growth with different OHP and 5-FU concentration after 72hs

| Group | | shRNA-C | shRNA-Pnn |
| --- | --- | --- | --- |
|  |  | Mean ± SE | Mean ± SE |
| OHP | Ctrl | 100 ± 0.81 | 87.25 ± 1.34* |
|  | OHP 0.02µM | 99.16 ± 3.88 | 91.51 ± 1.89* |
|  | OHP 0.1µM | 91.40 ± 4.46 | 69.13 ± 2.14* |
|  | OHP 0.5µM | 59.30 ± 3.64 | 53.36 ± 0.92* |
|  | OHP 1µM | 48.85 ± 1.55 | 43.41± 0.56* |
|  | OHP 3µM | 29.72 ± 3.01 | 26.80 ± 0.39 |
|  | OHP 10µM | 22.61 ± 1.66 | 23.71 ± 0.64 |
|  | OHP 30µM | 23.77 ± 0.80 | 26.18 ± 0.81 |
|  | OHP 100µM | 21.99 ± 1.12 | 20.55 ± 0.21 |
| 5-FU | Ctrl | 100 ± 2.12 | 84.97 ± 2.01* |
|  | 5-FU 0.02µM | 93.74 ± 5.41 | 98.36 ± 1.11* |
|  | 5-FU 0.1µM | 103.49 ± 2.31 | 99.60 ± 1.66* |
|  | 5-FU 0.5µM | 102.41 ± 2.87 | 79.23 ± 3.93* |
|  | 5-FU 1µM | 92.98 ± 6.02 | 61.42 ± 4.60* |
|  | 5-FU 3µM | 52.44 ± 2.90 | 48.03 ± 3.13* |
|  | 5-FU 10µM | 35.98 ± 0.73 | 33.34 ± 0.37* |
|  | 5-FU 30µM | 24.22 ± 0.52 | 25.35 ± 0.16 |
|  | 5-FU 100µM | 20.86 ± 0.62 | 25.69 ± 0.50 |

Data are expressed as percentage of the corresponding untreated control and reported as mean ± SE from at least three independent experiments. Statistical analyses were performed using two-way ANOVA. considering shRNA-Pnn silencing status and drug concentration as independent variables. followed by Tukey’s multiple comparisons post-hoc test. Significant differences were assessed between corresponding control and shRNA-Pnn groups at each OHP or 5-FU concentration. *p < 0.05 was considered statistically significant.
