# Supplementary material for: Pinin Modulation Restores Chemosensitivity in SW620 and HCT-116 Colon Cancer Cells
Source: Oncol Res. 2026 Oct 28;34(11):1. doi: 10.32604/or.2026.084313 (PMC13732370; doi:10.32604/or.2026.084313)
Supplement: Supplementary file 1 [file OncolRes-34-84313-s001.zip › Tables_S2_and_S3.docx]

**Table S2**

Inhibitory effects of siPnn respect to siCtrl on SW620 cells growth with different OHP and 5-FU concentration after 72hs

| Group | siCtrl | siPnn |
| --- | --- | --- |
|  | Mean ± SE | Mean ± SE |
| Ctrl | 100.00 ± 1.12 | 79.46 ± 5.33* |
| OHP 0.2µM | 64.69 ± 3.29 | 44.93 ± 3.15* |
| OHP 1µM | 53.02 ± 3.72 | 36.24 ± 1.81* |
| OHP 4µM | 45.63 ± 1.36 | 31.21 ± 2.14* |
| Ctrl | 100.00 ± 7.03 | 83.67 ± 7.51* |
| 5-FU 1µM | 76.15 ± 6.78 | 59.43 ± 7.65* |
| 5-FU 5µM | 68.44 ± 1.53 | 54.65 ± 2.47* |
| 5-FU 22µM | 57.27 ± 1.34 | 45.46 ± 1.31* |

Data are expressed as percentage of the corresponding untreated control and reported as mean ± SE from at least three independent experiments. Statistical analyses were performed using two-way ANOVA, considering shRNA-Pnn silencing status and drug concentration as independent variables, followed by Tukey’s multiple comparisons post-hoc test. Significant differences were assessed between corresponding control and shRNA-Pnn groups at each OHP or 5-FU concentration. *p < 0.05 was considered statistically significant.

**Table S3**

Inhibitory effects of stable SW620 shRNA-Pnn respect to shRNA-C cells line on growth with different OHP and 5-FU concentration after 72hs

| Group | shRNA-C | shRNA-Pnn |
| --- | --- | --- |
|  | Mean ± SE | Mean ± SE |
| Ctrl | 100.00 ± 3.13 | 84.69 ± 3.05 |
| OHP 0.2µM | 63.24 ± 1.01 | 45.45 ± 1.07* |
| OHP 1µM | 53.78 ± 1.23 | 38.37 ± 0.25* |
| OHP 4µM | 46.69 ± 0.42 | 25.92 ± 1.38* |
| Ctrl | 100.00 ± 1.01 | 92.94 ± 4.17 |
| 5-FU 1µM | 95.63 ± 0.67 | 86.38 ± 2.31* |
| 5-FU 5µM | 65.09 ± 1.27 | 54.92 ± 0.37* |
| 5-FU 22µM | 56.47 ± 0.27 | 47.02 ± 0.53* |

*p < 0.05 was considered statistically significant.
