# Supplementary material for: Pinin Modulation Restores Chemosensitivity in SW620 and HCT-116 Colon Cancer Cells
Source: Oncol Res. 2026 Oct 28;34(11):1. doi: 10.32604/or.2026.084313 (PMC13732370; doi:10.32604/or.2026.084313)
Supplement: Supplementary file 1 [file OncolRes-34-84313-s001.zip › Table_S1.docx]

Table S1: Primers of study genes and of 18S normalizer gene used in RT-PCR

| Genes | Forward (5’-3’) | Reverse (5’-3’) |
| --- | --- | --- |
| *ATM* | CTTGCTGACAATCATCACCAAGTTCGC | GTTTCAGGGTTCTCAGCACTATGGGAC |
| *PRKCZ* | CCGAGGAGACAGATGGAATTGCTTAC | CTTCAACCGCACCAGGAGAACCTTG |
| *HMGA1* | TCTTCCACCTGCTCCTTAGAGAAGGG | AGGTCTCTTAGGTGTTGGCACTTCGC |
| *MSTO1* | GTGTTCCTCTCCAGTTTCCATGGTTCAT | GGCAAAGCAACGTGTTCCAGAAGGC |
| *NDUFB9* | GCTTGTTTGATGAGAGCCCGGTTTGAAG | GCCAGTCATCTAAGCACCATTCTGGG |
| *C11orf65* | GGCAGAGCTTCTAGATGCTGCTGC | GCCACTATGATCCTCTTCCTGAAGATG |
| *POLA2* | GCTGTGATAGCAACGGGAAGCTGAAC | GTGGCACACCCTCGTAGAGTTTGG |
| *CRY1* | GGCTCCTCCAATGTGGGCATCAAC | CGTTCCTTTCCAAAGGGCTCAGAATC |
| *BAG1* | GAGTTGACCCTGAGTGAGGAAGCGA | AGGTCGTGCTTCTCATTGCTGTGGG |
| *PNN* | GAACAGAAGGCGGAACAAGAAGAGGG | TCATTGTGCTGATTACCTGTCTCCTCC |
| *18S* | CGGCTACCACATCCAAGGAA | GCTGGAATTACCGCGGCT |
